# Madagascar Flatidae (Hemiptera, Fulgoromorpha): state-of-the-art and research challenges

**DOI:** 10.3897/zookeys.319.4148

**Published:** 2013-07-30

**Authors:** Dariusz Świerczewski, Adam Stroiński

**Affiliations:** 1 Department of Zoology and Animal Ecology, Jan Długosz University, Al. Armii Krajowej 13/15, 42-201 Częstochowa, Poland; 2 Museum and Institute of Zoology PAS, Wilcza 64, 00-679 Warszawa, Poland

**Keywords:** Fulgoromorpha, Flatidae, taxonomy, research, history, Madagascar

## Abstract

The paper provides a historical review of the research on Flatidae in Madagascar and indicates future prospects. While the first two species of Madagascar Flatidae were described by Guérin-Méneville (1844), it was Signoret (1860) who made the first real attempt to enhance our knowledge of the Hemiptera fauna of Madagascar by describing several additional species. Over the following century and a half, several investigators have turned their attention to this group of insects, with the final number of species recorded for the island reaching 79. Despite this long history of research, it is evident that much still remains to be done. Detailed taxonomic research will allow the natural history of Madagascar and changes in the biological diversity of its endemic ecosystems to be better understood. This paper should be considered as an introduction to a complex study on the systematics and phylogeny of worldwide Flatidae planthoppers.

## Introduction

Flatidae constitute one of the largest families within planthoppers (Fulgoromorpha, Hemiptera) with 1446 species described in 299 genera and 12 tribes distributed worldwide (Bourgoin 2013). These phytophagous insects are highly diverse in terms of their colour and size (from 4.5 up to 32 mm), and are found on all continents, but are especially common and abundant in the tropics (O’Brien 2002). They are divided into two subfamilies – Flatinae and Flatoidinae, which, in most cases, can be easily distinguished from each other by the shape of the body. A greater number of Flatinae are flattened laterally, in contrast to Flatoidinae which hold their wings horizontally (O’Brien and Wilson 1985). About 20 species of Flatidae are regarded as serious pests of economically important crops such as coffee, tea, cacao, mango, citrus, apple and cherry (Wilson and O’Brien 1986). In Europe the only recognized pest is *Metcalfa pruinosa* (Say, 1830), a flatid species introduced into Italy from USA in 1973 (Arzone et al. 1987).

## Data resources

The data underpinning the analyses reported in this paper are deposited in the Dryad Data Repository at doi: 10.5061/dryad.d43j8

## Madagascar as a global biodiversity hotspot

Madagascar is one of eight important global biodiversity hotspots owing to its unique biota and the high level of threat to its natural habitats (McNeely et al. 1990, Myers et al. 2000, Ganzhorn et al. 2001). Being a part of the southern supercontinent Gondwana, it started to separate from Africa as a Madgascar-India block ca. 130 million years ago. At ca. 90 million years, India started breaking off from Madagascar and drifting northeastwards (Ali and Aitchison 2008). Despite Madagascar’s extreme isolation from India and its geographical proximity to Africa, a high proportion of the biota of the Madagascar region reveals some Asian affinities (Yoder and Nowak 2006). The suggested explanation of this might be the results of analyses obtained by Warren et al. (2010), which support the repeated existence of sizeable islands across the western Indian Ocean, reducing the distance of open ocean to be crossed.

Madagascar has evolved an incredible wealth of biodiversity, with thousands of species that can be found nowhere else on earth. On the one hand, this is due to its long isolation from all other landmasses (Storey et al. 1995), on the other, several alternative mechanisms have generated local endemism such as isolation within watersheds (Wilmé et al. 2006), adaptation along environmental gradients (Smith et al. 1997) and ecologically mediated postspeciation range shifts (Losos and Glor 2003). The relative importance of these mechanisms, both for Madagascar and globally, is still under discussion (Pearson and Raxworthy 2009, Vences et al. 2009). For example, of its estimated 12 000 plant species, nearly 10 000 are unique to Madagascar (Gautier and Goodman 2007). Among the fauna, vertebrates have been the most thoroughly studied Madagascan animals (Raxworthy and Nussbaum 1996, Raxworthy et al. 2002, Goodman and Ganzorn 2004). With regard to invertebrates, and insects in particular, the best known groups are butterflies (Torres et al. 2001, Zakharov et al. 2004), dung beetles (Wirta et al. 2008), minnow mayflies (Monaghan et al. 2005) and ants (Fisher 1997).

However, unsustainable use of natural resources such as wood for charcoal, bushmeat for protein supply and land for crop cultivations and cattle farming are having a profound impact on the Malagasy environment (Brown 2007). According to Kull (2012), an aerial photograph-based analysis of land-cover change of Madagascar’s high plateau in the latter half of the 20th century, based on a stratified rand sample of twenty eight sites, reveals a dramatic expansion of the cultural landscape of villages and agro-ecosystems into wetland and grassland environments. In response, significant environmental conservation efforts have been undertaken by the Malagasy government with international funding and technical support (Mittermeier et al. 2005). These environmental efforts in Madagascar have evolved over the past two decades from a Yellowstone model – transformation of large tracts of land into uninhabited, strictly protected areas, through the Integrated Conservation and Development Project (ICDP), which combines conservation of biological diversity with the social and economic needs of local people, to more recent initiatives that are the cutting edge of environmental innovation (Marcus and Kull 1999).

## Flatidae research in Madagascar: a historical account

The Flatidae fauna of Madagascar has intrigued natural historians for centuries and continues to attract more interest with time. The first description of flatid species dates back to the first half of the 19th century when French entomologist Félix Édouard Guérin-Méneville (1799–1874) gave a drawing and a short note on *Flata malgacha* (presently in genus *Flatida* White, 1846) and *Flatoides tortrix* in the work Iconographie du Règne Animal (Guérin-Méneville 1844). However, it was Victor Signoret (1816–1889) who made the first real attempt to enhance our knowledge of the Hemiptera fauna of Madagascar (Signoret 1860) by describing several additional species, in particular: *Nephesa antica*, *Nephesa suturalis*, *Phyllyphanta nivea*, *Elidiptera madagascariensis*, *Flatoides cicatricosus*, *Flatoides hyalinipennis*, *Flatoides sinuatus*, *Flatoides vicinus* (presently in the genera *Latois* Stål, 1866, *Flatopsis* Melichar, 1902, *Ulundia* Distant, 1910 and *Flatoidessa* Melichar, 1923). Moreover, the second half of 19th century was the realase of further papers by such eminent naturalists-hemipterologists as Carl Stål (1833–1878) and Ferdinand Karsch (1853–1936), which provided descriptions of additional representatives of Madagascan Flatidae (Stål 1866, Karsch 1890). Leopold Melichar (1856–1924), the foremost worker on Hemiptera at that time, made a substantial contribution with his two-part monograph of the world fauna of Flatidae by publishing re-descriptions of a number of species and describing a large number of new taxa, including several restricted just to Madagascar (Melichar 1901, 1902). This work was followed by a more concise volume of *Genera Insectorum* (Melichar 1923) also dedicated in part to Flatidae. Further species were described by Victor Lallemand (1880–1965) and Jacques Auber (1916–1995) (Lallemand 1933, 1950, Auber 1954, 1955). In 1956, Belgian entomologist Henri Synave (1921–1980) gave a short overview of Madagascan Flatinae, providing a key to all known species and simplified illustrations of some genital characters (Synave 1956). He also published a faunistic paper based on material collected in Madagascar during an expedition organized by The Natural History Museum in Basel, which was in fact the last one dedicated to Madagascan flatids (Synave 1966). Although John T. Medler (1914–2006), an outstanding researcher on the world fauna of Flatidae, later published two papers on west and southern African flatids (Medler 1988, 2001), neither of them contained any data referring to Madagascar. Summarizing, the Madagascan flatid fauna presently consists of 18 genera with 40 species of Flatinae and 12 genera with 39 species of Flatoidinae (Metcalf 1957, Stroiński and Świerczewski 2011, Stroiński and Świerczewski 2012a).

## Flatidae as a tool for biodiversity conservation: preliminary results

The accurate and rapid measurement of patterns of species richness, species turnover, and endemism is fundamental to current conservation efforts in Madagascar (Fisher 2007). One approach is to sample taxa that are ecologically important, but at the same time relatively easily collected, diverse at site and contain a high level of information for conservation planning. Flatidae meet these criteria and so might serve as an appropriate environmental indicator. According to Prof. Thierry Bourgoin from The Natural History Museum in Paris, this is the most abundant and easily collected group of the hemipterans in Madagascar (pers. comm.). Yet, the knowledge of the group is still very limited.

The wildlife of Madagascar is considered to be one of the most important human heritage resources of our time. The lack of appropriate tools for the recognition of biodiversity, which would also be useful in the protection of environment and natural resources, is a great disadvantage. Studies of Flatidae as a model group of phytophagous insects could be the solution for these needs, especially in relation to rare and endangered ecosystems. One of these is tapia woodlands – a short, endemic, sclerophyllous forest formation in Madagascar, with dominant canopy tree species *Uapaca bojeri* Baillon, 1874 or tapia in Malagasy. It can be found in four zones located in the central and southern part of the island, covering approximately 2600 km^2^ (Kull 2002). Tapia woodlands reveal strong adaptations to fire and are specifically human-shaped through controlled burning and selective cutting as they serve as a source of non-timber forest products for local residents (Kull 2003). The characteristic species for this ecosystem was recently described *Phleboterum tapiae* Świerczewski & Stroiński, 2012 (Świerczewski and Stroiński 2012b). Moreover, we discovered two new species *Flatopsis medleri* Świerczewski & Stroiński, 2011 and *Latois nigrofasciata* Świerczewski & Stroiński, 2012 confined exclusively to littoral forests (Świerczewski and Stroiński 2011, Świerczewski and Stroiński 2012a). Littoral forests have once formed an uninterrupted, narrow band along the entire length of Madagascar’s eastern coastline but as a result of anthropogenic activity their cover has been dramatically reduced to isolated, remnant fragments. Littoral forests are presently one of the smallest yet most diverse habitats in Madagascar. However, they are relatively unexplored and poorly documented ecosystems (De Gouvenain and Silander 2003). Approximately 13% of Madagascar’s total native flora can been found in these ecosystems and over 25% of the 1535 plant species known from littoral forests are endemic to this community (Consiglio et al. 2006). With respect to fauna, littoral forest are one of four major areas of ant endemism in Madagascar (Fisher and Girman 2000) Additionally, mountain flatid fauna seems quite rich and diverse, with endemic species and genera restricted to particular mountain massifs (work in prep.). An example can be the representatives of the genus *Urana* Melichar, 1902 – *Urana paradoxa* Melichar, 1902 and *Urana unica* Stroiński & Świerczewski, 2012, which are exclusively related to high altitude mountain rainforests (Stroiński and Świerczewski 2012b). Finally, we discovered the first representatives of the tribe Phantiini in Madagascar – *Soares testudinarius* Stroiński & Świerczewski, 2012 and *Madoxychara unicornis* Stroiński & Świerczewski, 2013 (Stroiński and Świerczewski 2012a, Stroiński and Świerczewski 2013). Phantiini is a small flatid tribe established by Melichar (1923) distributed worldwide and numbering 12 genera with 35 species. In Afrotropic it is represented by 6 genera and 11 species.

## Future research perspectives

Summarizing, despite a long history of observation and investigation, the state of knowledge of Madagascar’s Flatidae is deeply unsatisfactory. After gathering material from the most important entomological collections and two years of study we realized how much still remains to be discerned. In the area of systematics our research efforts are focused on redescriptions of all Madagascan Flatidae based on type specimens, descriptions of new species, complete synonymy, identification keys for genera and species, documentation of research in the form of drawings, SEM images and maps and a distribution catalogue of the species. Reinterpretation of characters formerly used for the identification of species and distinguishing higher taxa (tegmen venation and male genital structures) will also be included. Additionally, as a novelty, we will provide analysis of Flatidae female genital structures.

With respect to biodiversity and endemism, the basic parameters of the distribution, dispersal and zoogeographic status have yet to be understood. Furthermore, distinguishing species characteristic for particular vegetational formations is key to understanding the dynamics of endangered ecosystems. The revision will make it possible to estimate the level of endemism of Madagascan flatid fauna and its connections with the fauna of Oriental and Afrotropical regions. The project presented above is the first step in a complex study on the systematics and phylogeny of worldwide Flatidae planthoppers. It aims to clear up views on the species variability and diversity, create a modern classification system, and know in detail all the world genera and species. The goal of the long-term project is to complete the work on cladistic analysis, phylogeny and the evolutionary scenario proposal for the family.
